# The Complex of Cytochrome *f* and Plastocyanin from *Nostoc* sp. PCC 7119 is Highly Dynamic

**DOI:** 10.1002/cbic.201200073

**Published:** 2012-05-22

**Authors:** Sandra Scanu, Johannes Förster, Michela G Finiguerra, Maryam Hashemi Shabestari, Martina Huber, Marcellus Ubbink

**Affiliations:** [a] Institute of Chemistry, Leiden UniversityEinsteinweg 55, 2333 CC Leiden (The Netherlands); [b] Institute of Physics, Leiden UniversityNiels Bohrweg 2, 2333 CA Leiden (The Netherlands)

**Keywords:** NMR spectroscopy, photosynthesis, protein-protein interactions, redox proteins, spin label

## Abstract

Cytochrome *f* (Cyt *f*) and plastocyanin (Pc) form a highly transient complex as part of the photosynthetic redox chain. The complex from *Nostoc* sp. PCC 7119 was studied by NMR relaxation spectroscopy with the aim of determining the orientation of Pc relative to Cyt *f*. Chemical-shift-perturbation analysis showed that the presence of spin labels on the surface of Cyt *f* does not significantly affect the binding of Pc. The paramagnetic relaxation enhancement results are not consistent with a single orientation of Pc, thus indicating that multiple orientations must occur and suggesting that an encounter state represents a large fraction of the complex.

## Introduction

The concept of protein–protein complex formation is evolving towards a view in which an encounter state is in dynamic equilibrium with the well-defined specific complex.

The initial approach of the proteins and subsequent formation of the encounter state are thought to be mainly driven by long-range electrostatic forces, whereas the well-defined complex is stabilized by short-range interactions, like hydrogen bonds and van der Waals forces.[1] Until recently it was not possible to characterize the encounter state experimentally. However, several existing methods have been adapted for this purpose, like double-mutant cycles combined with measurements of association kinetics,[2] flash photolysis kinetics[3] and paramagnetic relaxation enhancement (PRE) NMR spectroscopy.[4^,^5] The first complex of electron transfer (ET) proteins characterized by this approach was that of cytochrome *c* (Cyt *c*) and cytochrome *c* peroxidase (CcP). The solution structure of this complex has been determined by PRE NMR,[6] which showed that Cyt *c* and CcP have the same relative orientation in the complex as in the crystal structure.[7] At the same time, the PRE data provided evidence of dynamics within the complex, thus suggesting that the encounter complex was significantly populated. By combining PRE data and Monte Carlo docking, the encounter state was visualized, and its fraction was established to be 30 %.[8] This approach opens the door for the characterization of encounter states in other transient redox complexes.[9]

Plastocyanin (Pc) and cytochrome *f* (Cyt *f*) form a redox complex in oxygenic photosynthesis. Pc shuttles electrons from Cyt *f* of the cytochrome *b*_6_*f* complex to P700 in photosystem I (PS I). The surface charge properties of Pc and Cyt *f*, which vary significantly between the different species, influence the relative orientation of the interaction partners in the well-defined complex. Two general orientations have been described, dubbed “side-on” and “head-on”. The side-on orientation has been observed in plant complexes.[10^,^11] The plant proteins exhibit a favorable electrostatic interaction because of the presence of negatively and positively charged amino acid patches on Pc and Cyt *f*, respectively. The patches align the long sides of Pc and Cyt *f*, thereby enabling rapid ET by bringing a hydrophobic patch on Pc close to the haem in Cyt *f.*[10] In the cyanobacterial complex from *Phormidium laminosum*, Pc approaches Cyt *f* “head-on”.[12] Within the complex, Pc is oriented perpendicular to the haem plane and only its hydrophobic patch participates in the interaction. Electrostatic interactions play a smaller role in *P. laminosum* than in plants,[13] although kinetics studies[14^,^15] suggested that charge interactions contribute to the formation of the encounter state. In the cyanobacterial complexes from *Nostoc* sp. PCC 7119[16] and *Prochlorothrix hollandica*,[17] where the charge distribution is reversed compared to that in plants, again the “side-on” orientation was observed. The solution models of the complexes have been determined by rigid-body docking of the structures of the individual proteins on the basis of binding chemical shift perturbations and intermolecular pseudocontact shifts (PCSs) of Pc nuclei induced by the paramagnetic, oxidized iron of Cyt *f*.[10] In the case of the *Nostoc* complex, site-directed mutagenesis studies on the influence of charges on the kinetics of complex formation highlighted how the loss of either positive charges on Pc[18] or negative charges on Cyt *f* [19] resulted in a decreased association rate constant. It could be shown that for Pc several charges are pivotal for the interaction.[18^,^19] On the other hand, the charges on Cyt *f* are more spread out over the surface, and no “hot spots” were identified in either *Nostoc*[19] or *P. laminosum,*[15] thus suggesting that the encounter state might have an important role in these complexes. To obtain independent restraints for the refinement of the well-defined state and to establish whether the encounter state is significantly populated, the Pc-Cyt *f* complex from *Nostoc* was studied by PRE NMR spectroscopy. The data cannot be described by the structure determined by PCSs alone, or indeed by any single structure, thus indicating that the encounter ensemble must represent quite a significant fraction of the complex.

## Results and Discussion

### Characterisation of MTS-tagged Cyt *f*

**Figure 1:**
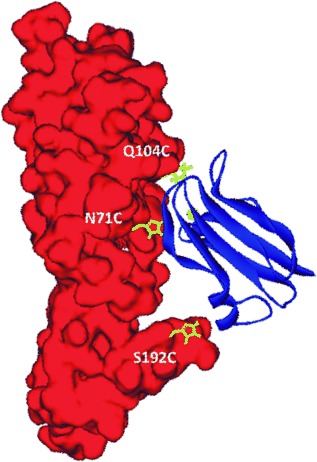
Locations of the spin labels on *Nostoc* sp. PCC 7119 Pc-Cyt *f* complex (PDB ID: 1TU2, model 1[16]). Pc is shown as ribbons with the copper as a sphere. Cyt *f* is shown as a surface. The spin labels were modeled on the structure (sticks). Images of molecular structures were made with Discovery Studio Visualizer 2.5 (Accelrys, San Diego, CA).

To study the complex of Cyt *f* and Pc with PRE NMR spectroscopy, three sites for probe attachment were selected. The positions of the mutations were selected on the basis of the solution structure of the wild-type complex as determined by NMR spectroscopy, on the basis of PCS and chemical shift perturbations.[16] The rationale of the work followed that of Volkov et al.[6] for the complex of CcP and Cyt *c*, that is, to obtain constraints for structure determination and to improve the precision of the solution structure that was based on PCS. Residues N71, Q104, and S192, which are located around the Pc binding site, were mutated to cysteine (Figure 1). In order to preserve the overall electrostatic potential in the complex only polar, uncharged amino acid residues were selected.

**Figure 2:**
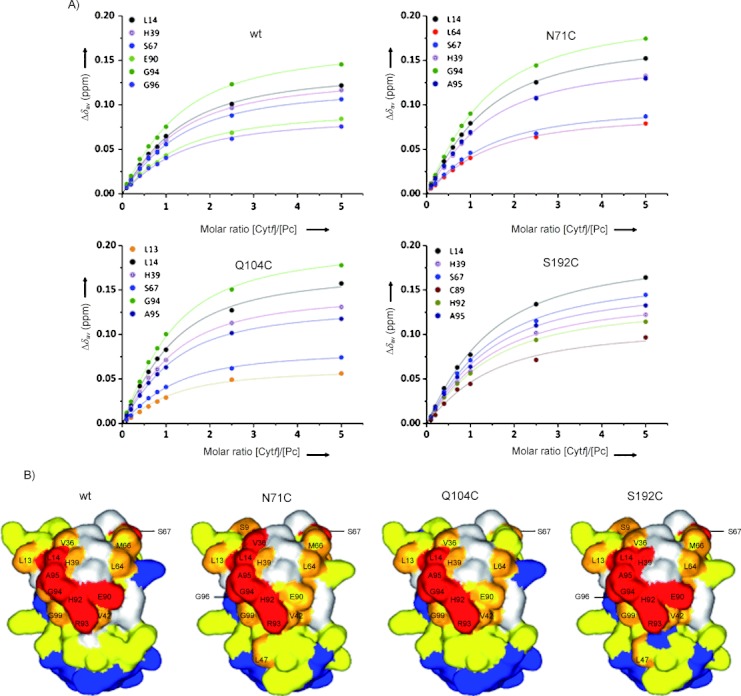
The interaction of *Nostoc* Zn-substituted Pc with wt Cyt *f* and MTS-conjugated variants. A) Binding curves for selected residues were fitted globally to a 1:1 binding model [Eq. (2)]. B) Chemical shift perturbation maps of Zn-substituted Pc in the presence of wild-type and MTS-conjugated Cyt *f*, color-coded on a surface model of Pc (PDB ID: 2CJ3). Red, Δ*δ*_avg_≥0.10 ppm; orange, Δ*δ*_avg_≥0.05 ppm; yellow, Δ*δ*_avg_≥0.02 ppm; blue, Δ*δ*_avg_<0.02 ppm. Prolines and residues with overlapping resonances are shown in gray.

^15^N-enriched Pc was produced in a cytoplasmic expression system (see the Experimental Section) with a Zn^II^ ion in the copper binding site to eliminate the paramagnetic effect of Cu^II^ and possible interference from electron transfer reactions.[20] To establish whether the introduction of a probe interferes with the Pc–Cyt *f* interaction, chemical shift analyses were carried out for all variants. First, Zn-substituted [^15^N]Pc was titrated with wild-type Cyt *f*, and HSQC spectra were acquired at each titration point. The binding constant was obtained by fitting the chemical shift perturbation curves for the most affected amide groups (Figure 2 A), thereby yielding a *K*_d_ of 8(3)×10^−5^ m (estimated experimental error in parentheses), similar to the reported values of 3.8(0.1)×10^−5^ m for Cu-Pc[21] and 6.2(0.9)×10^−5^ m for Cd-substituted Pc.[21]

Also the binding map is similar, with the largest perturbations observed for residues L14, G94, and A95, corresponding to the hydrophobic interaction patch, and H92 and R93 of the basic patch (Figure 2 B, wt).

Similar titrations of Pc and the cysteine mutants of Cyt *f* conjugated with the diamagnetic control label (1-acetoxy-2,2,5,5-tetramethyl-δ-3-pyrroline-3-methyl)methanethiosulfonate (MTS) yielded the dissociation constants listed in Table 1. The binding curves and maps are shown in Figure 2. Clearly, the mutations and attachment of MTS have very little effect on the affinity and the binding map.

**Table 1:** Dissociation constants of the complexes formed between Zn*-*substituted Pc and wt or MTS-conjugated Cyt *f*.

Cyt *f* mutant	*K*_d_ [10^−5^ m][a]	Cyt *f* mutant	*K*_d_ [10^−5^ m][a]
wt	8 (3)	Q104C-MTS	3 (1)
N71C-MTS	4 (1)	S192C-MTS	4 (1)

[a] Estimated experimental errors are indicated in parentheses.

### Paramagnetic relaxation enhancements of Pc nuclei

**Figure 3:**
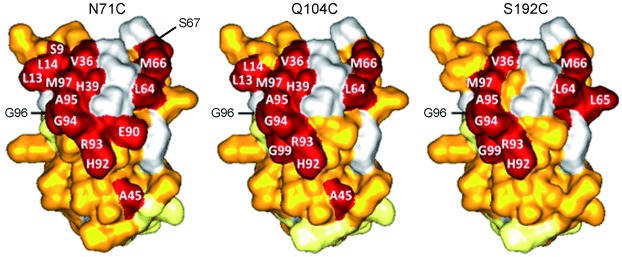
PRE maps of Zn-substituted Pc bound to MTSL-conjugated Cyt *f*, color-coded on a surface model of Pc (PDB ID: 2CJ3) according to the three classes of restraints defined for the docking: residues with *I*_p_/*I*_d_<0.10 (red), 0.10<*I*_p_/*I*_d_<0.95 (orange) and *I*_p_/*I*_d_>0.95 (yellow). Prolines and residues with overlapping resonances are shown in grey.

The aim of this study was to gather distance restraints from PREs to refine the published solution structure. The residues selected for mutation to cysteine and tagging with the spin label are located around binding site for Pc on Cyt *f* in the solution structure model. The spin labels at these positions were thus expected to yield PRE of nuclei on different sides of Pc. For this purpose, a spin label (1-oxyl-2,2,5,5-tetramethyl-δ-3-pyrroline-3-methyl)methanethiosulfonate (MTSL) was attached to the three Cyt *f* mutants, and the tagged protein was added to Pc at a Cyt *f*/Pc molar ratio of 0.3:1. Under these conditions, the fraction of bound Pc was 24 %. Large PREs were observed already at this ratio for numerous Pc amide groups for each variant of Cyt *f*-MTSL, as illustrated in Figure 3.

Some resonances were broadened beyond detection. It is clear from the Cyt *f* titrations that Pc binding is in the fast exchange regime, so an observed PRE is a weighted average of free Pc (no PRE), the encounter state (the AB* ensemble) and the final complex, AB, Equation (1). 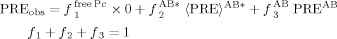
(1)

**Figure 4:**
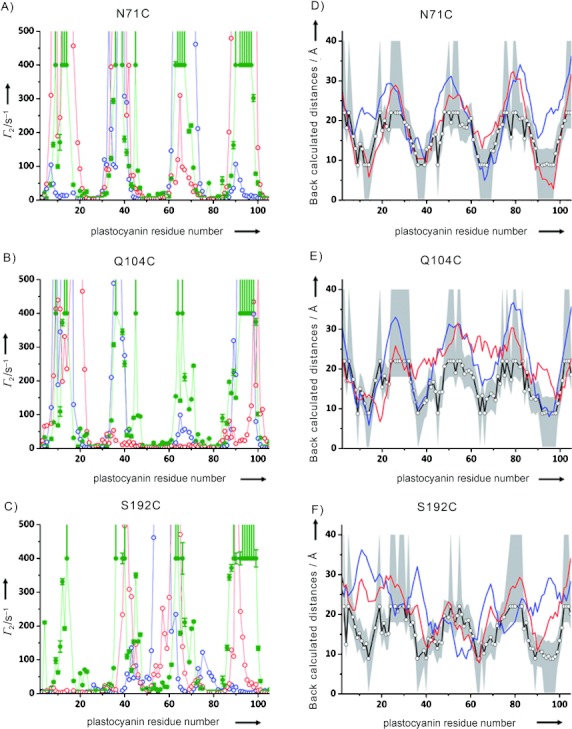
A)–C) Observed and predicted PRE values for amide protons in Pc bound to MTSL-conjugated Cyt *f* variants. The observed PREs were extrapolated to represent the 100 % bound state of Pc (green dots). PREs at 400 s^−1^ represent lower limits. The PREs calculated from the NMR solution structure based on PCS (PDB ID: 1TU2, model 1[16]) are shown as blue symbols. The PREs calculated from the NMR solution structure based on PREs are shown as red symbols. D)–F) Experimental and back-calculated distances between Pc amide protons and MTSL-conjugated Cyt *f* variants. The white circles and black line represent the distances calculated from the experimental PREs (which were extrapolated to 100 % bound Pc); gray area: error margins. The distances derived from the NMR solution structure based on PREs are shown as a red line. The distances derived from the NMR solution structure based on PCS (PDB ID: 1TU2, model 1[16]) are shown as a blue line.

**Figure 5:**
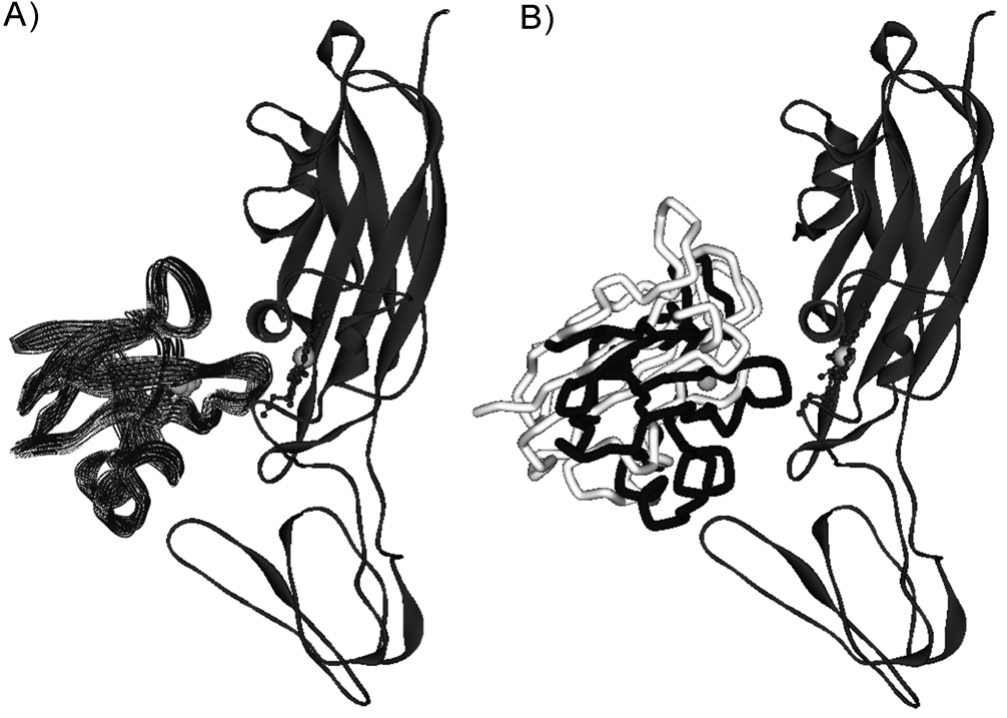
A) Model of the Pc–Cyt *f* complex obtained with PREs restraints. Cyt *f* is shown in ribbons and Pc as Cα trace. The ten lowest-energy structures are shown. B) Overlay of Pc molecules from the NMR solution structure based on PREs (black Cα trace) and the NMR solution structure based on PCS (PDB ID: 1TU2, model 1,[16] light gray).

The fraction of free Pc (*f*_1_) is 0.76, and that of bound Pc (*f*_2_+*f*_3_) is 0.24. By dividing the observed PRE by 0.24, the PRE for 100 % bound Pc is obtained. These extrapolated PREs are plotted in Figure 4 (green symbols) against the Pc residue number. Strikingly, the patterns are qualitatively similar for the three spin label positions, thus indicating that the same patches of Pc are strongly affected. When the fraction of AB* is neglected (*f*_2_≈0), the PRE can be predicted from the model of the final complex. By using model 1 from PDB ID: 1TU2, the PRE^AB^ values were predicted for each amide in Pc (Figure 4, blue symbols). Clearly, the model alone cannot account for the observed PREs. Also, docking calculations were performed with distances derived from the PREs as restraints. Apart from a van der Waals repulsion function (to avoid steric collisions) no other interactions were included. The ensemble of the ten best structures is shown in Figure 5 and compared with the model based on PCS. In both cases, Pc is bound in the region close to the haem, but the orientation differs. However, also the PRE-based model alone cannot account for the observed PREs and the back calculated distances (Figure 4, red symbols in the left panel, red line in the right panel).

Thus, it can be concluded that a single orientation is insufficient to describe the Pc–Cyt *f* complex. It is now well-established that PREs are very sensitive to lowly populated states. The poor fit between the PRE data and the modeled structure indicates that other orientations of Pc within the complex contribute to the PRE data. This conclusion is supported by the similarity of the PRE maps in Figure 3 for the Cyt *f* variants. If the Pc were in a single orientation in the complex, different patches of Pc residues would have been affected by PRE, because the spin labels are located around the binding site (e.g., red symbols in Figure 4). Yet, for each spin label position, the same Pc surface region is affected by PREs; this also matches the side with the largest chemical shift perturbations. These observations suggest that Pc samples a large area of the surface of Cyt *f* with its hydrophobic patch. Our results are in accord with kinetic experiments that indicated that the interaction site of Pc depends on a few specific residues, whereas for Cyt *f* the residues relevant in the association are more spread over the protein surface.[18^,^19] The formation of the encounter complex reduces the dimensionality of the diffusional search for the binding site that enables rapid ET.[1] It has been suggested that the population balance between the encounter state and the well-defined state depends on whether rapid ET can occur in encounter-state orientations.[8] In complexes of small proteins the redox centers can get sufficiently close for ET in many of the protein orientations, but in larger complexes fast ET can only occur through certain areas of the protein surface, thus requiring the formation of a well-defined complex. Both Cyt f and Pc have elongated shapes, and the metal ion is close to only a small part of the surface. Thus, it seems likely that a degree of specificity in the interaction is required for this complex to be active. Earlier studies suggested that the degree of dynamics varies between Cyt *f*–Pc complexes. Those of *P. laminosum*[12] and *Pr. hollandica*[17] appeared to be particularly dynamic. From the data presented in this study, the fraction of the encounter complex cannot be established, but it is clear that it is significant, even though the complex in *Nostoc* spp. would be categorized as well-defined given the earlier NMR data, that is the intermolecular PCS from the Cyt *f* haem to Pc.[16] Both PCS and PRE are sensitive to minor states populating the encounter complex, in the case in which the minor state experiences a much stronger paramagnetic effect than the major state. In the opposite case (where the major orientation is most affected by paramagnetism), the presence of minor states might well go unnoticed, because it only leads to a small reduction in the observed effect. Here, the PCSs are large, particular for the major state, which is close to the paramagnetic haem, whereas the PREs will be dominated by those orientations that bring the nucleus close to the spin label; thus, the PREs describe better the combination of the final complex and the encounter ensemble. Therefore, a good fit of the PCS data was obtained with a single structure in the study of Diaz et al.,[16] while the same structure is insufficient to account for all PREs here. More extensive spin labeling covering a large area of the surface will enable a detailed description of the encounter complex, as was shown for the ET complex of Cyt *c* and CcP.[8] Such experiments are underway.

## Experimental Section

**Protein production and purification:** The plasmid pEAP-WT, containing the gene encoding Pc in *Nostoc* sp. PCC 7119, was kindly provided by Prof. Dr. Miguel A. De la Rosa (University of Seville). The leader sequence (34 amino acids) was removed in order to achieve cytoplasmic expression of the mature Pc (as defined in Un*i*Prot entry O52830). An N-terminal Met residue was added to initiate translation. This gene was obtained by PCR with the following primers.

FWD: 5′-ctgtgcaa**ccatgg**aaacatacacagtaaaactaggtagcg-3′

REV: 5′-ctgtgcaa**ctcgag**ttagccggcgacagtgattttacc-3′.

NcoI and XhoI restriction sites were introduced in the forward and reverse primers, respectively, and are indicated with bold letters. The former incorporates the ATG codon for the initiation Met residue. The amplified gene and the vector pET28a were doubly digested with these enzymes before ligation. The construct (pSS01) was verified by DNA sequencing.

Uniformly ^15^N-labeled Pc was produced in *E. coli* BL21 freshly transformed with pSS01. A single colony was inoculated in lysogeny broth (LB, 2 mL) with kanamycin (25 mg L^−1^) and cultured until the OD_600_ reached 0.6. From this, an aliquot (50 μL) was inoculated into ^15^N M9 minimal medium (50 mL), in which [^15^N]-NH_4_Cl (0.3 g L^−1^) was the only source of nitrogen, with kanamycin (25 mg L^−1^), and the culture was incubated overnight. An aliquot (5 mL) was transferred into ^15^N minimal medium (0.5 L), and incubated until the OD_600_ reached 0.6. All cultures were incubated at 37 °C with shaking at 250 rpm. Expression of the gene encoding Pc was induced with isopropyl β-d-1-thiogalactopyranoside (IPTG, 1 mm) at 22 °C. The cells were harvested after 20 h by centrifugation using a Fiberlite* F10-6x500y rotor in a Sorvall RC6+ centrifuge at 6400 *g* for 20 min. The pellet was resuspended in sodium phosphate buffer (NaP_i_; 10 mL, 1 mm, pH 7). Phenylmethylsulfonyl fluoride (PMSF, 1 mm), DNase (0.2 mg mL^−1^), and ZnCl_2_ (250 μm) were then added. Cells were lysed by using a French Press. The cell lysate was separated by ultracentrifugation using a Kontron TFT 55.38 rotor in a Centrikon T-1170 ultracentrifuge at 120 000 *g* for 30 min at 4 °C, and the supernatant was dialyzed overnight against NaP_i_ (1 mm, pH 7) with ZnCl_2_ (25 μm). The solution was cleared by ultracentrifugation and loaded on a carboxymethyl (CM) cellulose Sephadex Fast Flow column (Amersham Biosciences) equilibrated with NaP_i_ (1 mm, pH 7). The elution was carried out with a gradient of NaP_i_ (1–25 mm, pH 7). The fractions containing Pc were loaded once again on the column and eluted under the same conditions. The concentration of the protein was determined by absorbance spectroscopy (ε_280_=5 mm^−1^ cm^−1^). The yield of pure protein was 10 mg per liter of culture. The absence of Cu was verified by UV/Vis spectroscopy (absence of the characteristic band at 595 nm under oxidizing conditions). The presence of Zn was verified by atomic absorption spectroscopy.

The pEAF-WT plasmid, containing the gene of the soluble domain (residues 1–254) of *Nostoc* sp. PCC7119 Cyt *f*, was kindly provided by Prof. Dr. Miguel A. De la Rosa (University of Seville). pEAF-WT was used as template to obtain Cyt *f* mutants. Mutations to cysteine were introduced by using the QuikChange Site-Directed Mutagenesis kit (Stratagene). The primers used for the mutations at the positions N71 and Q104 were as described before.[22] The primers employed for the introduction of a cysteine at position S192 were:

FWD: 5′-ggcgaagatggttgcgttaaatatttagt**c**gacatc-3′

REV: 5′-gatgtc**g**actaaatatttaacgcaaccatcttcgcc-3′

For S192C, a silent mutation (bold) introduced an extra SalI restriction site, located close to the 3′ end of the forward primer. Codon-changing mutations are underlined. The mutant genes were verified by DNA sequencing.

Truncated Cyt *f* was produced in *E. coli* MV1190, (D(*lac*-*pro*AB), *thi*, *sup*E, D(*srl*-*rec*A) 306::Tn10 (tet^r^) [F′:*tra*D36, *pro*AB+, *lac*I^q^Z?M15]) or mutant plasmids and co-transformed with pEC86,[23] which contains a cassette for *c*-type cytochrome overexpression. A single colony was inoculated into LB (50 mL) containing chloramphenicol (20 mg L^−1^) and ampicillin (100 mg L^−1^), and cultured (37 °C, 250 rpm, 5–6 h). Culture (5 mL) was used to inoculate LB (1.7 L) with the same antibiotics in a 2 L Erlenmeyer flask. The culture was incubated (25 °C, 150 rpm, 20 h), then further chloramphenicol (20 mg L^−1^) and ampicillin (100 mg L^−1^) were added. After further incubation (2 h), gene expression was induced with IPTG (1 mm). The cells were harvested 96 h after induction. The purification of the protein was performed as previously reported.[18] Dithiothreitol (DTT, 3 mm) was added to the buffers during the purification to prevent the dimerisation of the cysteine mutants. It was removed immediately before spin labeling by buffer exchange on a PD10 column (GE Healthcare), equilibrated with 2-(*N*-morpholino) ethanesulfonic acid (MES) buffer (20 mm, pH 6). The ferrous form of the Cyf *f* cysteine mutants (20–80 μm) was used for attachment of MTS or MTSL. A 20-fold molar excess of MTS(L) was added, and the solution was incubated for one hour on ice. A 100-fold molar excess of K_3_Fe(CN)_6_ was then added to oxidize the haem iron and prevent reduction of the nitroxyl group or the disulfide bridge by the ferrous heme.

The sample was concentrated by ultrafiltration to a volume of 0.5 mL and loaded on a Superdex75 gel filtration column (GE Healthcare) equilibrated with MES buffer (20 mm, pH 6). The fractions containing MTS(L)-Cyt *f* were concentrated and the buffer was exchanged by ultrafiltration to MES (20 mm, pH 6), K_3_Fe(CN)_6_ (0.5 mm). The attachment of the spin label was verified by mass spectrometry, and the presence of the nitroxyl radical was checked by EPR spectroscopy. The concentration of the protein was determined by absorbance spectroscopy (ε_556_=31.5 mm^−1^ cm^−1^ for ferrous Cyt *f*).

**NMR:** All NMR samples contained MES (20 mm, pH 6) and 6 % D_2_O for lock. Cyt *f* was kept in the ferric state by addition of K_3_Fe(CN)_6_ (50 μm). The pH of the sample was adjusted with HCl (0.5 m) and NaOH (0.5 m). For the chemical shifts perturbation experiments Cyt *f* was titrated into Zn-substituted [^15^N]Pc (50 μm). Spectra were recorded at multiple Cyt *f*/Pc molar ratios (0.1, 0.2, 0.4, 0.6, 0.8, 1.0, 2.5, and 5.0). Samples for PRE measurements contained Cyt *f* (66 μm) labeled with either MTS or MTSL and Zn-substituted [^15^N]Pc (200 μm). All NMR spectra were recorded at 298 K on an Avance III 600 MHz spectrometer equipped with a TCI-Z-GRAD CryoProbe (Bruker). The ^1^H,^15^N HSQC spectra were acquired with 1024 and 80 complex points in the direct and indirect dimensions, respectively.

**Data analysis:** The NMR spectra were processed with NmrPipe[24] and analyzed with CcpNMR.[25] The assignments for the Zn–[^15^N]Pc amide resonances were kindly provided by Dr. Mathias A. S. Hass. The assignments for residues K6 and V29 could not be made because of overlap of the corresponding peaks in the HSQC spectra. The chemical shift perturbations (Δ*δ*_bind_) of Pc resonances resulting from complex formation with Cyt *f* were plotted against the molar ratio of Cyt *f*/Pc (*R*). Note that the perturbations include both the effect of binding and the PCS caused by the ferric heme iron, in all samples. The entire perturbation was used in the analysis; the PCS was not used separately in this study.

The corresponding titration curves were fitted in OriginLab 8.1 (http://www.originlab.com) with a non-linear least square fit to a 1:1 binding model, Equation (2).[26]

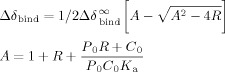
(2)

Here 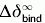 is the chemical shift perturbation for 100 % bound Pc, *P*_0_ is the starting concentration of Pc and *C*_0_ is the stock concentration of Cyt *f*. A global fit with a single binding constant (*K*_a_*=K*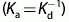) for the data of several residues was used.

The binding maps were obtained by extrapolation of the Δ*δ*_bind_ values at the 5:1 Cyt*f*/Pc molar ratio of all residues to 100 % bound Pc when using the *K*_d_. These extrapolated perturbations were averaged for the nitrogen (Δδ_N_) and hydrogen (Δ*δ*_H_) atoms of each amide, thereby yielding Δ*δ*_avg_, according to Equation (3): 
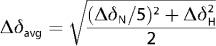
(3)

The PREs were determined according to the procedure of Battiste and Wagner.[27] The intensity ratio *I*_p_/*I*_d_ of the Pc resonances in the presence of MTSL-Cyt *f* (*I*_p_) and MTS-Cyt *f* (*I*_d_) were normalized by dividing them by the average value of the ten largest *I*_p_/*I*_d_ values (1.13 for N71C and Q104C; 1.06 for S192C). The PRE (*Γ*_2_) values were calculated according to the formula [Eq. (4)]: 
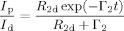
(4)

The transverse relaxation rates in the diamagnetic sample (*R*_2d_) were calculated from the line width at half height obtained from a Lorentzian peak fit in the direct dimension, by using MestReC (http://www.metsrelab.com). The symbol *t* denotes the time for transverse relaxation during the pulse sequence (9 ms).

**Structure calculations:** The PREs were converted into distances for structure calculations by using Equation (5): 
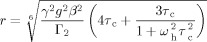
(5)

Here *r* is the distance between the oxygen atom of MTSL and the Pc amide proton, *γ* is the proton gyromagnetic ratio, *g* is the electronic g-factor, *β* is the Bohr magneton, *ω*_h_ is the Larmor frequency of the proton and *τ*_c_ is the rotational correlation time of the MTSL oxygen-proton vector. *τ*_c_ was taken to be 30 ns on the basis of the HYDRONMR[28] prediction of the rotational correlation time for the Pc–Cyt *f* complex. In the docking procedure this value gave rise to the lowest energy structures in comparison with *τ*_c_ values of 20, 25, 35, and 40 ns.

Three classes of restraints were included in the calculations. 1) For residues with *I*_p_/*I*_d_ < 0.1 (including those for which the resonances disappeared from the spectrum), the upper bound was set to 14 Å. 2) For residues with *I*_p_/*I*_d_ > 0.95, the lower bound was set to 22 Å. 3) For residues with *I*_p_/*I*_d_ between 0.1 and 0.95, the distances calculated from Equation (5) were used with upper and lower bounds of 4 Å. The structure calculations were done in Xplor-NIH.[29] Cyt *f* and Pc were both considered as rigid bodies, the coordinates of Cyt *f* were fixed, and Pc was allowed to move in a restrained rigid-body molecular dynamics calculation. The structure of the soluble domain of Cyt *f* used for the calculation was taken from the crystal structure of the cytochrome *b*_6_*f* complex from *Nostoc* sp. PCC 7120 (PDB ID: 2ZT9).[30] The amino acidic sequences of Cyt *f* from *Nostoc* sp. PCC 7120 and sp. PCC 7119 are identical. Mutations and spin labels were modeled on the structure of Cyt *f*. Four conformations were used to represent the mobility of the spin label,[31] and the distances to the Pc nuclei were r^−6^ averaged for these MTSL conformers. The structure of Pc was taken from PDB ID: 2CJ3. At each cycle, Pc was placed at a random position and the protein was docked as rigid body on the basis of only experimental restraints and a van der Waals repulsion function to avoid steric collision. Two hundred approaches were performed, thereby yielding 155 structures with restraint energies below a given threshold. The ten lowest-energy structures were selected, and they showed an average rmsd of 0.83 Å to the mean structure.
